# Humans need only 200 ms to generate posture-specific muscle activation patterns for successful vertical jumps in reaction to an auditory trigger

**DOI:** 10.3389/fspor.2023.1123335

**Published:** 2023-05-17

**Authors:** Maarten F. Bobbert, Axel S. Koopman

**Affiliations:** Department of Human Movement Sciences, Faculty of Behavioural and Movement Sciences, Vrije Universiteit Amsterdam, Amsterdam Movement Sciences, Amsterdam, Netherlands

**Keywords:** initial posture, reaction time, optimal control, muscle coordination pattern, EMG

## Abstract

**Introduction:**

It is currently unknown how the central nervous system controls ballistic whole-body movements like vertical jumps. Here we set out to study the time frame of generating muscle activation patterns for maximum-effort jumps from different initial postures.

**Methods:**

We had ten healthy male participants make a slow countermovement from an upright position and initiate a maximal vertical jump as soon as possible following an auditory trigger. The trigger was produced when hip height dropped below one of three preselected values, unknown in advance to the participant, so that the participant was uncertain about the posture from which to initiate the jump. Furthermore, we determined the ensuing bottom postures reached during jumps, and from these postures had the participants perform maximum-effort squat jumps in two conditions: whenever they felt ready, or as soon as possible following an auditory trigger. Kinematics and ground reaction forces were measured, and electromyograms were collected from gluteus maximus, biceps femoris, rectus femoris, vastus lateralis, gastrocnemius and soleus. For each muscle, we detected activation onsets, as well as reaction times defined as the delay between trigger onset and activation onset.

**Results:**

In the jumps preceded by a slow countermovement, the posture from which to initiate the jump was unknown before trigger onset. Nevertheless, in these jumps, posture-specific muscle activation patterns were already released within 200 ms after trigger onset and reaction times were not longer and jump heights not less than in squat jumps from corresponding bottom postures.

**Discussion:**

Our findings suggest that the generation of muscle activation patterns for jumping does not start before trigger onset and requires only about 200 ms.

## 1. Introduction

Seemingly without mental effort, humans perform very complex ballistic whole-body movements. The most impressive performances occur in sports. Take, for example, jumping in volleyball in order to form a defensive block. The offensive actions of the players of the attacking team dictate where a defender should make the jump, often with very limited preparation time. Furthermore, due to the dynamics of the game, the defender often has to jump from postures other than preferred. Nevertheless, defenders jump high and generally block successfully. The motor performance in these complex dynamic situations fills spectators with awe and envy, and is perplexing from an engineering perspective: How does the central nervous system manage to control—with so little preparation time and such high precision—a multi-link inverted pendulum with highly nonlinear actuators and limited speed of signal transport?

Motor control theories have primarily been developed for visually guided limb movements to targets in external space. Many investigators raised the question of how the brain translates the spatial information specified by the target into muscle activation patterns that will bring the limb along a specific trajectory to the desired location (e.g., 1–3). It has been proposed that after determination of the location of the target in space, and planning of a desired trajectory to bring the endpoint of the limb to the target, the following transformations occur: an inverse kinematic transformation yields the joint angle trajectories corresponding to the desired endpoint motion, and an inverse dynamics transformation first yields the joint torques to generate the joint angle trajectories, then the muscle forces to generate the joint torques, and finally the muscle stimulations to generate the muscle forces, all as a function of time (1, 4). More recent theories, such as the optimal control framework, do not rely on intermediate goals such as desired trajectories; all available resources are used to pursue the high-level movement goal (5). The path of the hand during a goal-directed arm movement is the result of a control policy, which itself is a result of minimization of a cost or effort (6).

It is currently unknown whether ballistic whole-body movements are controlled in a similar way as goal-directed limb movements. In vertical jumping, it seems reasonable to define the high-level movement goal in terms of the height reached by the center of mass. The maximal achievable height is defined by the properties of the musculoskeletal system, and the actual height reached depends on the stimulation of the muscles over time (7). Hence, reaching maximal jump height in itself requires optimization of the muscle stimulation pattern; introducing any extra cost will be detrimental for the high-level movement goal. In several studies, musculoskeletal simulation models have been developed, and dynamic optimization or optimal control theory has been used to find stimulation-time inputs that maximized the height reached by the center of mass, typically by very time-consuming computation (e.g., 8–14). It has been shown that the resulting kinematic, kinetic, and muscle-coordination patterns are very similar to those observed when humans jump to their maximum achievable heights (e.g., 8–12, 14). However, establishing that the human nervous system obtains the same solutions as optimization algorithms in simulation studies does not say anything about how the central nervous system achieves this (15–17). Also, it does not tell us anything about timescales; for example, if optimal solutions are used, are they computed in the reaction time of a movement task *de novo*? (6).

When it comes to the control of vertical jumping, ideas on how it might be organized have ensued from several experimental and simulation studies. It has been shown that visco-elastic properties of muscles play an important role in counteracting kinematic perturbations and simplify the control of jumping (18). In a simulation model, the stabilizing role of muscle properties could even be exploited to make successful jumps from a range of starting postures without adapting control, albeit that this inevitably caused jump heights to be lower than maximal (19). However, when humans perform maximum-effort jumps from various equilibrium postures, they do actually adjust their muscle activation pattern (9, 10). By simulating jumps with a model comprising four body segments and six Hill-type muscles, it has been shown that the adjustments are near-optimal, i.e., they cause the ensuing jump height to be near-maximum (9, 10). We are not aware of any other studies that focused on the control of jumping.

With respect to the question of how the central nervous system adjusts muscle activation patterns to initial postures in jumping, the only thing that can be said for certain is that it cannot be based on feedback generated during the motion itself. For example, when participants performed squat jumps from different initial squat depths (9), the plantar flexors were the first muscles to become activated when the center of mass (CoM) was initially high, and were the last muscles to become activated when CoM was initially low. This reversal in onsets cannot be based on feedback generated during the execution of the movement, for the simple reason that the execution of the motion, and hence the generation of (re)afference, does not start until the first muscle is activated. It is attractive to say, then, that the adjustments must be largely “prepared” or “pre-programmed” using proprioceptive information available in the initial posture. But what does this “preparation” or “pre-programming” entail? In computer simulation of jumping, it is quite a challenge to find the optimal muscle activation pattern that drives a multi-link inverted pendulum actuated by highly nonlinear muscle-tendon complexes to its maximum jump height (11–14). Could the human nervous system be engaged in any preparatory mental simulation with an internal model of the musculoskeletal system (20, 21) to find the optimal muscle activation pattern that maximizes jump height from the given initial posture? At this moment we do not have a clue.

In the experimental studies referred to above, participants made jumps from equilibrium initial postures and were free to initiate the push-off whenever they felt ready. Hence, they were able to use all the time they needed to prepare muscle activation patterns. But how much time do humans actually need to prepare muscle activation patterns for jumping? What happens when the time available for preparation of motor commands is reduced? Do participants resort to control solutions that are not adapted to the initial posture and, if so, does this lead to a reduction of performance? Or do they still manage to generate muscle activation patterns that are adapted to the initial posture? Here we tried to find answers to such questions by introducing uncertainty about the posture from which to initiate vertical jumps and limiting the time available for preparation of muscle activation patterns. We will show that within 200 ms following an auditory trigger humans already release posture-specific muscle activation patterns for successful jumps.

## 2. Materials and methods

### 2.1. Outline of experimental procedures

Ten physically fit and healthy male participants participated in the study. They all had several years of practice in sports that involved jumping. The experiments were conducted in accordance with the Declaration of Helsinki, the study was approved by the local ethics committee, and all procedures were carried out with the adequate understanding and written consent of the participants. Mean (SD) characteristics of the group of participants were: age 22 (2) yr., height 1.87 (0.07) m, and body mass 74.9 (7.1) kg.

The outline of the experiment was as follows. Starting from upright standing, participants made a slow countermovement, and initiated a vertical jump as soon and as high as possible following an auditory trigger. The trigger occurred when the height of the hip during the countermovement dropped below one of three preselected values, unknown in advance to the participant. Hence, the participants were uncertain about the posture from with the jump was to be initiated. We determined the bottom postures from which the participants ended up jumping (i.e., the postures at which the vertical velocity of the center of mass switched from downward to upward) and had the participants perform maximum-effort squat jumps from these postures. In one condition they did so whenever they felt ready to do so, and in a second condition they did so as soon as possible following an auditory trigger. During all jumps, participants held their arms behind their backs and their hands interlocked.

With the experimental conditions outlined above we set out to answer four specific questions: (1) Does jumping as soon as possible following a trigger, rather than at a freely chosen instant, have a detrimental effect on jump height? (2) Does uncertainty about the posture from which to jump have a detrimental effect on jump height? (3) Is the muscle activation pattern adjusted to the posture from which to jump if this posture is unknown until the trigger occurs? (4) Does uncertainty about the posture from which to jump increase reaction time?

**Figure 1:**
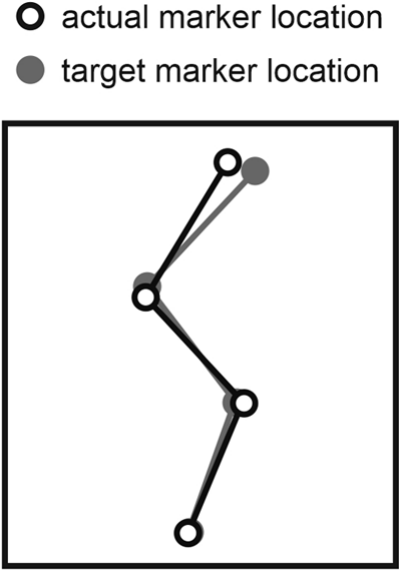
Matching of target posture using online kinematic feedback. In squat jumps, the initial posture was set by having the participant match a target posture that was previously extracted at the bottom of a jump preceded by a slow countermovement.

In the experiment, we measured ground reaction forces with a force-platform, monitored sagittal-plane positional data of anatomical landmarks, and recorded electromyograms from six muscles of the right lower extremity, as will be detailed later on. The experimental session for each participant started with the determination of hip height (height of a marker on greater trochanter) while the participant was standing upright with heels on the ground. Subsequently kinematic and ground reaction force data were captured in two equilibrium positions on tiptoes, one upright and one in which the hips were flexed and the upper body was oriented horizontally; these data were used to determine the location of the center of mass of the upper body relative to the other kinematic markers on the upper body (22). We then instructed the participant to perform two types of maximum-effort jumps (the naming convention of which is explained below and in the Glossary), and allowed him to practice each type. Jumps of the first type were jumps following a slow countermovement initiated from upright standing, during which an auditory trigger occurred (CT); the participant was instructed to perform the countermovement (C) slowly, taking at least 1 s to reach the preferred deepest squat position, and to initiate the push-off as soon as possible following the auditory trigger (T). The trigger occurred during the countermovement when the height of the hip dropped below 90% (CTH), 75% (CTM) or 67% (CTL) of its value in upright standing. Jumps of the second type were squat jump (S), with the instruction to make no preparatory downward movement. For the squat jumps we had two conditions; in one the participant was instructed to initiate the push-off as soon as possible following an auditory trigger (ST), and in the other the participant was free (F) to initiate the push-off whenever he felt ready (SF). Squat jumps were executed from an initial posture that matched a target initial posture. This target initial posture could be either the participant's preferred initial squatted posture (SFP) or the posture at the instant that the hip reached its lowest height (henceforth referred to as “bottom”) during a particular CT jump (SFH, SFM, SFL). The kinematic marker positions of the target posture were shown on a computer screen together with the online positions of the markers, and the participant matched the latter with the former in the equilibrium initial phase of the jump (Figure 1).

After a participant had practiced the performance of the different types of jumps the actual experiment started and the participant performed a total of 31 jumps, with consecutive jumps being separated by two minutes of rest. The first two were squat jumps from the preferred position, one without trigger (SFP) and one with trigger (STP). Subsequently the participant performed in random order three CTH, three CTM and three CTL jumps. In each of these, the posture at the instant that the hip reached its lowest height was extracted. From the latter postures, we determined for CTH, CTM and CTL separately the average posture to be used as target initial posture for corresponding ST jumps. The participant then performed, in random order, three STH, three SFH, three STM, three SFM, three STL and three SFL jumps. Finally, he performed again one SFP and one STP jump, the heights of which were compared to the corresponding jumps at the start of the experiment to check whether any fatigue had occurred. Jump height, defined as the difference between the height of CoM at the apex of the jump and the height of CoM when the participant was standing upright with heels on the ground, was calculated from the positional data. Details on the setting of the initial postures and on data collection and processing, as well as the specific questions that we addressed and how we addressed them, are provided below.

### 2.2. Collection and processing of data

Ground reaction forces were measured using a force platform (Kistler 9281B, Kistler Instruments Corp., Amherst, NY). The output signals of the platform were amplified (Kistler 9865E charge amplifier, Kistler Instruments Corp., Amherst, NY), sampled at 200 Hz, and processed to determine the fore-aft and vertical components of the reaction force and the location of the center of pressure.

For kinematic analysis, infrared light emitting diodes were placed on the right side of the body at the acromion, greater trochanter, lateral femoral epicondyle, lateral malleolus, and fifth metatarsophalangeal joint. Together, these markers defined the positions of four body segments: HAT (head, arms and trunk), thighs, shanks and feet. During jumping, the markers were monitored in 3D using two OPTOTRAK 3020 units (Northern Digital, Waterloo, Ontario) operating at 200 Hz. Only sagittal plane projections were used in this study. During CT jumps, the trigger occurred when the online monitored height of the hip marker dropped below the selected value.

Off-line, the time histories of marker positions were smoothed using a zero-lag 4th-order low-pass Butterworth filter with a cut-off frequency of 6 Hz, which seemed adequate for the purpose of this study since we did not calculate any derivatives. The locations of the mass centers of thighs, shanks and feet were estimated by combining the landmark coordinates with results of cadaver measurements presented in the literature (23). As described elsewhere (22) we determined the location of the center of mass of HAT relative to the two markers defining this segment from kinematic and kinetic data obtained from the two equilibrium postures specified earlier. With this information, the location of CoM was calculated in all other body configurations found during jumping, and used to calculate jump height.

We used electromyography to determine the time course of the neural input to the muscles (24). To record electromyograms (EMG) from the muscles of the right leg, pairs of Ag/AgCl surface electrodes (Medicotest, blue sensor, type: N-00-S) were applied to the skin overlying gluteus maximus, biceps femoris (caput longum), rectus femoris, vastus lateralis, gastrocnemius (caput mediale) and soleus, all according to procedures advocated by SENIAM (25). The EMG-signals were amplified and sampled at 1000 Hz (Porti-17t, Twente Medical Systems). Off-line, they were high-pass filtered at 7 Hz to remove any possible movement artefacts, full-wave rectified, and smoothed using a causal digital 10 Hz cutoff low-pass Butterworth filter (filter function in MATLAB R2011b, The MathWorks Inc., Natick, MA, 2000), to yield smoothed rectified EMG (*srEMG*) (26, 27). For each muscle in each participant, *srEMG* was subsequently normalized for the maximal value observed for that muscle across all trials of that participant to yield *nsrEMG*.

**Figure 2:**
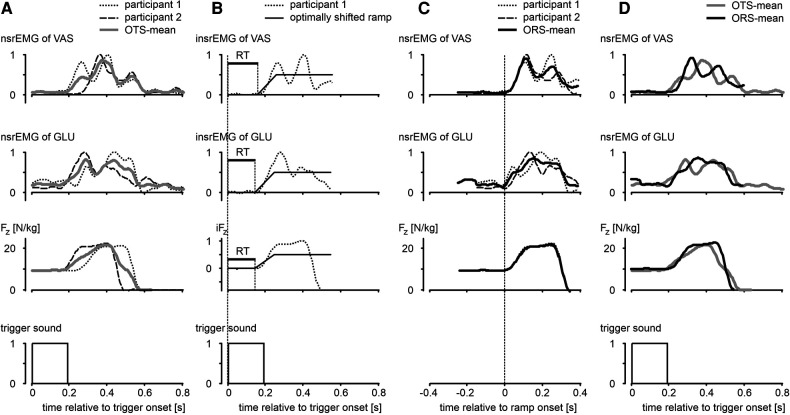
Illustration of methods used to determine reaction times (RT) and average curves. **Column A**: examples of smoothed rectified EMG-signals of m. vastus lateralis (VAS) and m. gluteus maximus (GLU) normalized for the maximal value across all trials (*nsrEMG*), and vertical component of the ground reaction force (*F_z_*), all as function of time relative to onset of an auditory trigger. OTS-mean: average of individual curves synchronized relative to trigger onset. **Column B**: signals after subtraction of the average value during the first 100 ms following trigger onset, and subsequent normalization for the maximum value. We searched and found the best fit between these intermediate signals (prefix “i”) and a ramp signal increasing from 0 to 0.5 in 100 ms, by shifting the onset of the ramp. Reaction time (RT, horizontal bar in panels) was defined as the time between trigger onset and the onset of the optimally shifted ramp. **Column C**: determination of ORS-mean curves after synchronization of individual curves for onset of ramp. **Column D**: comparison of ORS-mean curves after addition of the corresponding average RT to the time axis, with OTS-mean curves. Compared to OTS-mean curves, ORS-mean curves gave a better representation of the rising phase of the original curves.

For all jumps with a trigger (CT and ST) we detected onsets of *nsrEMG*, from which we subtracted the onset of the trigger to obtain reaction times (RT). Detecting onsets of *nsrEMG* was a challenge because muscle activity was not always stationary; after all, in the squat jumps the participants were balancing, and in the other jumps they were slowly moving downwards, sometimes on the balls of the feet and toes. We tried out various methods to detect onsets and settled on the method illustrated in Figure 2. We first subtracted from each original *nsrEMG*-signal (examples in Figure 2, column A) the average value during the first 100 ms following the trigger, and normalized the result for the maximum value (Figure 2, column B). We then searched and found the best fit (lowest Root Mean Square) between this signal and a ramp signal that increased from 0 to 0.5 in 100 ms, by shifting the onset of the ramp. The onset of the optimally shifted ramp was used as the onset of the *nsrEMG*-signal. Admittedly, there was some variation in the slopes of the *nsrEMG*-signals of different muscles and different participants; it may be noted, for example, that the slope of the *nsrEMG*-signals was higher than that of the ramp (Figure 2, column B). We also explored a method in which both the onset and the slope of the ramp were optimized, but this produced ostensibly erroneous detections in a couple of trials. The method that we ended up using was fast and robust, and with the chosen fixed slope of the ramp shown in Figure 2 the onset of the optimally shifted ramp was always very close to the onset of the *nsrEMG*-signals that we would select by visual inspection. The method just described was also used to detect the onset of the vertical component of the ground reaction force (*F_z_*, third row Figure 2).

**Figure 3:**
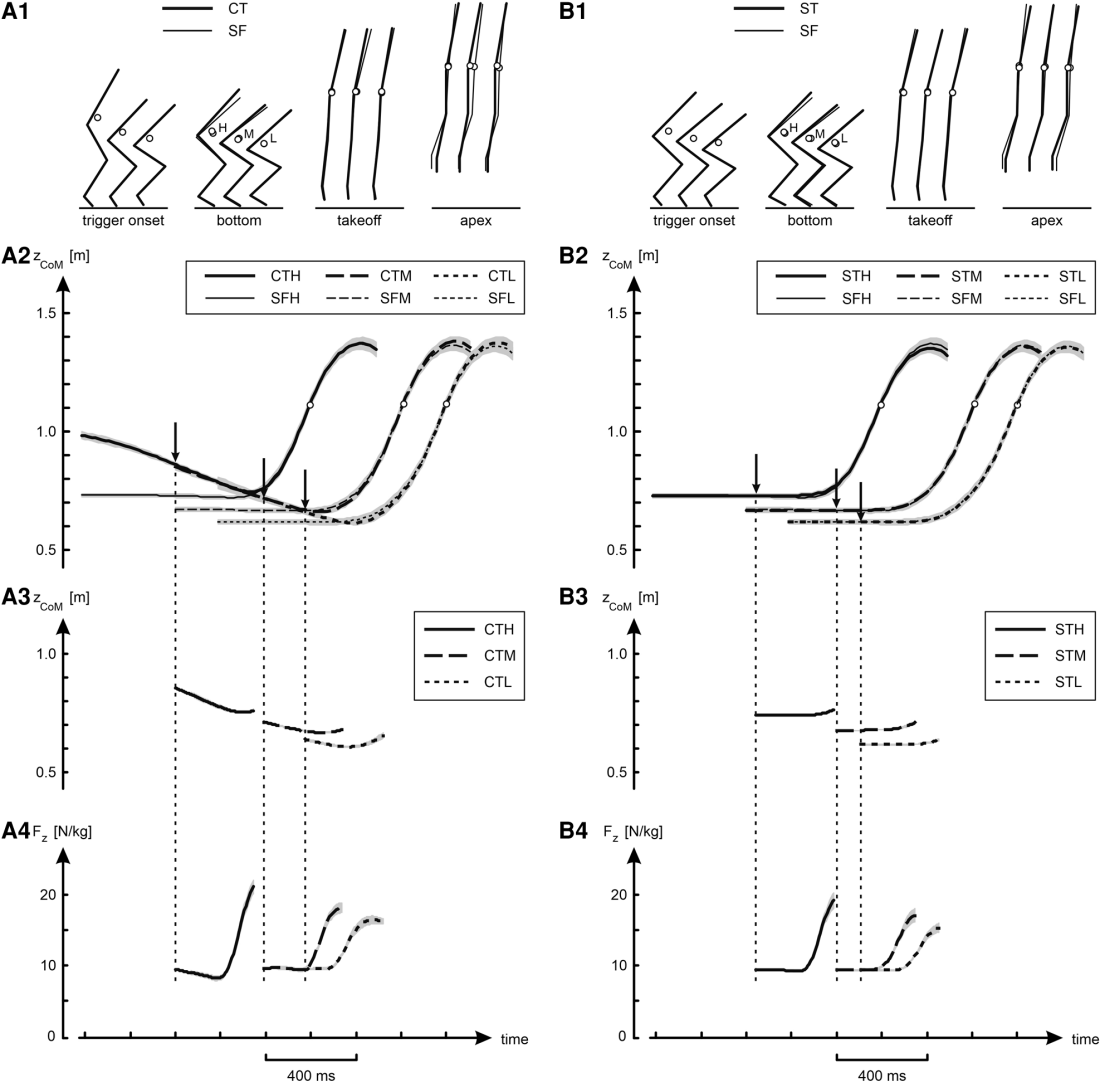
Kinematic and kinetic results of jumps in various conditions. **Panel A1** shows average stick diagrams at selected instants during the jumps: at trigger onset, at the bottom of the jump, at takeoff, and at the apex of the jump. The open circles in the stick diagrams represent CoM, and H, M and L stand for High, Medium and Low, respectively. **Panel A2** shows curves of the height of CoM (*z_CoM_*) as a function of time, obtained by averaging per condition individual curves synchronized at takeoff; shaded areas represent SEM. The three curves for the jumps with slow countermovement (CTH, CTM and CTL) have been aligned such that they coincided in the initial countermovement phase, and the arrows indicate the average trigger onset. The three curves for the free squat jumps (SFH, SFM and SFL) were aligned with their corresponding countermovement curve at takeoff. **Panels A3** and **Panel A4** show for the jumps preceded by a slow countermovement curves for respectively *z_CoM_* and *F_z_* as a function of time, obtained by averaging per condition individual curves synchronized at the onset of the trigger (OTS-curves), as explained in Figure 2. **Column B** presents the same information as **column A**, but this time comparing the squat jumps with trigger (STH, STM and STL) to the corresponding free squat jumps (SFH, SFM and SFL).

In addition to presenting statistics based on RT, we will present graphs of mean signals over time with the Standard Error of the Mean (28) indicated by shaded areas. Because subjects converge towards an extended body posture during the push-off, a good impression of the overall kinematic differences among the jump types could be obtained by averaging kinematic signals after synchronizing them at take-off (e.g., Figure 3, Panel A2). However, we were also interested in the changes following the auditory trigger in quickly varying force signals and electromyographic signals; because RT could differ among participants (e.g., Figure 2, column A) and among trials within a participant, determination of mean signals over time that preserved the shape of the individual signals was a challenge. Simply averaging individual signals at fixed times after synchronization at the onset of the trigger (OTS) yielded a mean signal with a rising phase that poorly represented the shape of the original signals (OTS-mean, Figure 2). The latter shape was preserved relatively well when signals were averaged after synchronization of onsets of the ramp (ORS-mean, Figure 2, column C). In the remainder of this paper, we will present ORS-mean curves (with SEM as shaded areas) with the corresponding average RT added to the time axis so that the curves of different signals were aligned at trigger onset (ORS-mean, Figure 2, column D). Also, we will present ORS-mean curves with time expressed relative to the average onset of *nsrEMG* of gluteus maximus; in a previous study it was shown that choosing the onset of *nsrEMG* of gluteus maximus as reference resulted in the smallest variation in the other *nsrEMG*-onsets across conditions (10).

### 2.3. Specific questions addressed and statistics

To determine whether jumping as soon as possible following a trigger, rather than at a freely chosen instant, had a detrimental effect on jump height, we compared jump height achieved in ST-jumps with that achieved in the corresponding SF-jumps. To determine whether uncertainty about the posture from which to jump had a detrimental effect on jump height, we compared the height achieved in CT-jumps with the height achieved in the corresponding ST-jumps and SF-jumps. To determine whether the muscle activation pattern was adjusted to the posture from which to jump, even if this posture was unknown until the trigger occurred, we compared the onsets of *nsrEMG* within CT- and ST-jumps across the three postures. To determine whether uncertainty about the posture from which to jump increased reaction time, we compared the onsets of *nsrEMG* among CT- and ST-jumps. All comparisons were made using a General Linear Model ANOVA for repeated measures (Statistical Package for the Social Sciences for Windows; SPSS Inc., Chicago, IL). When a significant F-value was found in a particular ANOVA, *post hoc* pair wise comparisons of means were made, using Šidák correction where appropriate. The level of significance for all tests was 0.05. In tables, we will report per variable per condition a mean and a standard deviation (SD), where the latter was calculated over ten values, each of which was the mean over the trials of one participant in a given condition.

## 3. Results

All participants were able to follow the instructions in the different conditions and perform successful jumps. Jump height was 36.6 (5.4) cm in the SFP jump at the start of the experiment and 36.1 (7.1) cm in the SFP jump at the end of the experiment; the difference was not statistically significant. For the STP jumps the corresponding values were 35.2 (5.9) cm and 35.8 (7.0) cm and again the difference was not statistically significant. These findings led us to conclude that no fatigue occurred during the experiment.

Figure 3 and Table 1 present an overview of the kinematic and kinetic findings. Panel A2 of Figure 3 shows height of CoM (*z_CoM_*) as a function of time. The curves for the different jumps preceded by a slow countermovement (CTH, CTM and CTL) have been aligned such that they coincided at the beginning of the countermovement, and the arrows indicate the average onset of the trigger. Each of the curves for the free squat jumps conditions (SFH, SFM and SFL) was aligned at takeoff with the curve of the corresponding jump preceded by a slow countermovement. Panel A1 shows average stick diagrams at selected instants during the jumps, with the open circles representing CoM. In agreement with our intention, the initial posture for the squat jumps was quite close to the posture at the bottom of the corresponding jump preceded by a countermovement and, not surprisingly, the same was true for *z_CoM_* (Table 1). Also, takeoff positions (Panel A1 in Figure 3) and the corresponding *z_CoM_* at takeoff (Table 1) were virtually identical, from which it may be deduced that also the jumps preceded by a slow countermovement, during which the trigger occurred at a posture unknown in advance, were well-behaved. Panels A3 and A4 in Figure 3 show for the jumps with slow countermovement mean OTS-curves for *z_CoM_* and *F_z_*, respectively. Column B in Figure 3 shows that the squat jumps with trigger (STH, STM and STL) were almost identical to their corresponding free jumps (SFH, SFM and SFL).

**Table 1:** Center of mass height at selected instants during the countermovement jumps (C) and squat jumps (S). Values presented are means (SD) for all participants (*n* = 10) in the initial posture *z_CoM,ini_*, at the bottom of the jump (*z_CoM,min_*), at takeoff (*z_CoM,to_*), and at the apex of the jump (*z_CoM,max_*), all relative to the height of the center of mass in upright standing. The effect of performing jumps as soon as possible following a trigger sound (T), rather than at a freely chosen instant (F), was investigated by comparing results in ST-jumps, in which the trigger sound was present, with those in SF-jumps, in which the participants jumped whenever they felt ready. The effect of uncertainty about the posture from which to jump was investigated by comparing results in CT-jumps, in which the posture was unknown until trigger onset, with those in ST-jumps in which it was known long before trigger onset. Initial postures for ST-jumps were derived from the postures reached at the bottom of the CT-jumps which could be High (H), Medium (M) or Low (L). Participants also performed squat jumps from the initial posture that they preferred (*P*).

Variable	Condition	*P*	H	M	L
*z_CoM,ini_* [cm]	CT		−0.7 (0.8)	−0.4 (1.3)	−0.6 (0.9)
	ST	−38.1 (7.0)	−29.3 (3.5)	−35.4 (4.0)	−40.3 (3.9)
	SF	−37.3 (6.9)	−29.0 (3.8)	−35.2 (4.0)	−40.3 (3.8)
*z_CoM,min_* [cm]	CT		−28.4 (4.2)	−36.7 (4.4)	−41.1 (3.8)
	ST	−38.4 (6.8)	−29.6 (3.3)	−35.6 (3.9)	−40.5 (3.9)^*^
	SF	−39.2 (7.2)	−31.1 (3.6)	−36.4 (3.6)	−40.8 (3.8)
*z_CoM,to_* [cm]	CT		9.2 (3.3)	9.8 (2.8)	9.7 (3.4)
	ST	9.5 (3.2)	9.3 (3.0)	9.4 (3.0)	9.3 (2.7)
	SF	9.7 (2.8)	9.6 (3.0)	9.6 (2.8)	9.4 (2.8)
*z_CoM,max_* [cm]	CT		35.0 (6.1)	35.8 (5.5)	35.4 (6.7)^#^
	ST	35.5 (6.3)	33.1 (5.8)	33.8 (6.3)	33.6 (6.3)^*^
	SF	36.3 (6.1)	35.1 (7.1)	34.4 (6.5)	34.0 (7.0)

^*^ Indicates a main effect (*p* < 0.05) of performing jumps as soon as possible following a trigger sound (ST), rather than at a freely chosen instant (SF).

^#^ Indicates a main effect (*p* < 0.05) of uncertainty about the posture from which to make the jump (CT) compared to when there was no such uncertainty (ST).

Our first question was: Does jumping as soon as possible following a trigger, rather than at a freely chosen instant, have a detrimental effect on jump height? We found that ST-jumps were lower than the corresponding SF-jumps: on average the difference was −0.9 (1.6) cm (Table 1), with the main effect being statistically significant [F(1,9) = 8.02, *p* < 0.05]. The difference in height ranged from −1.9 cm between STH and SFH to −0.4 cm between STL and SFL. In contrast to the instruction, the participants could not resist making a very small countermovement in the squat jumps, the amplitude of which was bigger in the SF-jumps than in the ST-jumps. It amounted to 1.9, 2.1, 1.2 and 0.5 cm on average in SFP, SFH, SFM and SFL, respectively, but only to 0.3, 0.3, 0.2 and 0.2 cm on average in STP, STH, STM and STL, respectively (Table 1, see also Panels A2 and A4 of Figure 3).

Our second question was: Does uncertainty about the posture from which to jump have a detrimental effect on jump height? We observed that CT-jumps were not lower but higher, on average by 1.9 (2.0) cm, than the corresponding ST-jumps [Table 1, F(1,9) = 11.9, *p* < 0.05]. As a matter of fact, CT-jumps were on average even higher than the corresponding SF-jumps (Panels A1 and A2 in Figure 3), on average by 0.9 cm (Table 1), but this difference was not statistically significant. This unexpected outcome may have to do with the performance-enhancing effect of the slow countermovement. In regular countermovement jumps, this effect is on the order of 3.4 cm (22); the countermovement allows for building up active state and force before the start of the concentric push off, and hence more work can be produced than during a squat jump where active state and force have to be built up during the upward motion (29). In regular countermovement jumps *F_z_* at the start of the upward movement is about 2.5 times body weight [Figure 8 in (22)], reflecting that muscle forces are much higher than in a squat jump from the same bottom posture. In the current study, the countermovement was performed at about half the preferred speed, but *F_z_* at the start of the upward movement was still considerable: 2.3 (0.5), 1.9 (0.7) and 1.8 (0.7) times body weight in CTH, CTM and CTL, respectively.

**Figure 4:**
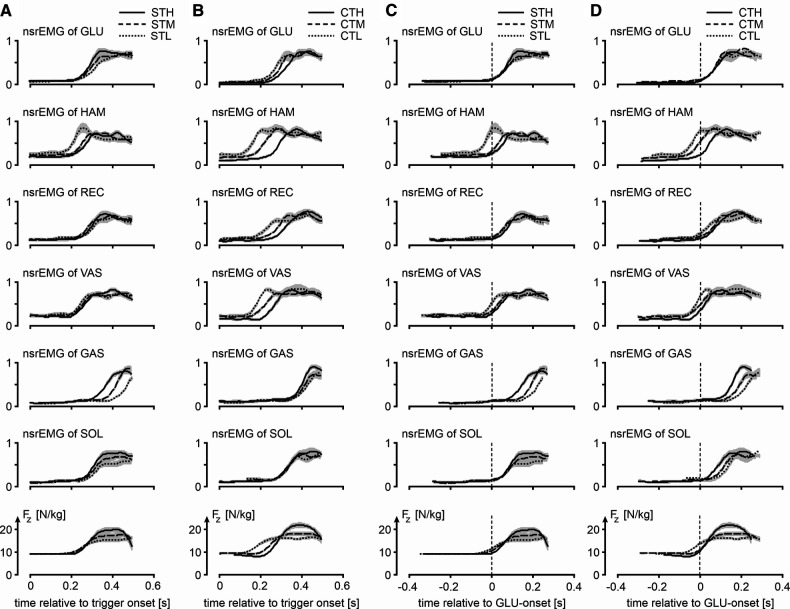
Muscle activity and vertical ground reactions force of jumps in various conditions. **Column A** and **column B** show for the squat jumps following a trigger (STH, STM and STL) and jumps preceded by a slow countermovement during which the trigger occurred (CTH, CTM and CTL), respectively, mean ORS-curves (similar to **column D** of Figure 2) of normalized smoothed rectified EMG-signals (*nsrEMG*) of the different muscles and the vertical component of the ground reaction force (*F_z_*), with time expressed relative to trigger onset (t = 0). Shaded areas represent SEM. **Column C** and **column D** show the same ORS-curves but with time expressed relative to the onset of *nsrEMG* of GLU (t = 0). Muscle abbreviations are: GLUteus maximus, HAMstrings (biceps femoris, long head), RECtus femoris, VAStus lateralis, GAStrocnemius, and SOLeus.

Our third question was: Is the muscle activation pattern dependent on the posture from which to jump even if this posture is unknown until the trigger occurs? Figure 4 presents for all ST-jumps (column C) and CT jumps (column D) ORS-mean curves of *F_z_* and *nsrEMG* of all muscles, with time expressed relative to the average onset of *nsrEMG* of gluteus maximus. Onsets of the various signals relative to the onset of *nsrEMG* of gluteus maximus are presented in Table 2. We found a statistically significant interaction effect between condition and muscle [F(8) = 6.5, *p* < 0.05, note that gluteus maximus onset relative to itself was left out of the analysis] and further analyzed main effects of condition per muscle. We found that the onset of *nsrEMG* of m. gastrocnemius relative to the onset of *nsrEMG* of gluteus maximus occurred later as *z_CoM_* at the bottom posture was lower, both in ST-jumps [F(2,9) = 6.6, *p* < 0.05] and in CT-jumps (F(2,9 = 12.1, *p* < 0.05). We also found that the onset of *nsrEMG* of hamstrings relative to the onset of *nsrEMG* of gluteus maximus occurred earlier as *z_CoM_* at the bottom posture was lower in ST-jumps [F(2,9) = 4.3, *p* < 0.05] and in CT-jumps [F(2,9) = 9.1, *p* < 0.05].

**Table 2:** Onsets of smoothed rectified EMG (*srEMG*) of various muscles relative to *srEMG*-onset of gluteus maximus. Postures reached at the bottom of the triggered countermovement jumps (CT) and squat jumps (ST) could be High (H), Medium (M) or Low (L). Mean values (SD) are presented for the group of participants (*n* = 10). Muscle names are HAMstrings (biceps femoris, long head), RECtus femoris, VAStus lateralis, GAStrocnemius, and SOLeus.

Muscle	Condition	H	M	L
HAM	CT	−40 (31)	−85 (73)	−107 (47)^*^
	ST	−15 (51)	−18 (95)	−73 (85)^*^
REC	CT	8 (63)	−6 (80)	−30 (116)
	ST	7 (45)	−0 (69)	−12 (113)
VAS	CT	−51 (22)	−71 (51)	−80 (53)
	ST	−26 (25)	−37 (55)	−68 (71)
GAS	CT	78 (91)	119 (119)	155 (90)^*^
	ST	61 (76)	103 (95)	133 (120)^*^
SOL	CT	13 (75)	55 (130)	72 (133)
	ST	15 (68)	24 (91)	7 (105)

^*^ Indicates a main effect of posture from which to jump on *srEMG*-onset (*p* < 0.05).

Our fourth question was: Does uncertainty about the posture from which to jump increase reaction time? It was already obvious from Panel A4 in Figure 3 that *F_z_* starts to increase 200 ms or less after trigger onset. This is also obvious from the left half of Figure 4, showing for ST-jumps (column A) and CT jumps (column B) ORS-mean curves of *F_z_* and *nsrEMG* of all muscles, with the corresponding average RT added to the time axis so that the curves of different signals were aligned at trigger onset. Onsets of the various signals relative to trigger onset are presented in Table 3. In CTH and STH the vasti were the first muscles to become active with the same average RT of 213 ms. In CTM the hamstrings were the first muscles to become active after an average RT of 156 ms, whereas in STM the vasti were the first muscles to become active after an average RT of 207 ms. In CTL and STL the hamstrings were the first muscles to become active after an average RT of 111 ms in CTL and 193 ms in STL. Thus, there was no indication that reaction time was increased by uncertainty about the posture from which to jump; if anything, reaction time was less in the jumps in which there was uncertainty about this posture.

**Table 3:** Reaction times, defined as onsets of smoothed rectified EMG (*srEMG*) relative to onset of trigger. Postures reached at the bottom of the triggered countermovement jumps (CT) and squat jumps (ST) could be High (H), Medium (M) or Low (L). Mean values (SD) are presented for the group of participants (*n* = 10). Muscle names are GLUteus maximus, HAMstrings (biceps femoris, long head), RECtus femoris, VAStus lateralis, GAStrocnemius, and SOLeus.

Muscle	Condition	H	M	L
GLU	CT	264 (51)	241 (57)	218 (56)
	ST	238 (33)	244 (46)	266 (63)
HAM	CT	223 (59)	156 (63)	111 (67)^*^
	ST	223 (55)	226 (84)	193 (89)^*^
REC	CT	271 (52)	235 (98)	188 (134)
	ST	246 (63)	244 (71)	254 (138)
VAS	CT	213 (39)	170 (49)	138 (32)^*^
	ST	213 (25)	207 (46)	198 (55)^*^
GAS	CT	342 (83)	360 (119)	373 (79)
	ST	299 (92)	347 (121)	399 (134)
SOL	CT	277 (88)	296 (117)	290 (113)
	ST	254 (82)	268 (103)	273 (112)

^*^ Indicates a main effect of posture from which to jump on reaction time (*p* < 0.05).

## 4. Discussion

In jumping, the central nervous system succeeds in projecting our body—a multi-link inverted pendulum with highly nonlinear actuators and limited velocity of signal transport—to near maximum height with little preparation time and high precision. It has previously been shown that when humans start maximum-effort jumps from various equilibrium postures without time pressure, they adjust their muscle activation pattern to the initial posture (9, 10). Here we asked participants to perform maximum-effort jumps as soon as possible following a trigger from initial postures that were unknown beforehand. We found that posture-specific muscle activation patterns were released within 200 ms following trigger onset, and that they resulted in successful performance. Below, we will elaborate on our findings and discuss what they teach us about the generation of muscle stimulation patterns for jumping.

Our first finding was that performing maximum-effort squat jumps from a given initial posture as soon as possible following a trigger rather than at a freely chosen instant, had a detrimental effect on jump height (*p* < 0.05), ranging from 2 cm between STH and SFH to 0.4 cm between STL and SFL (Table 1). Since the initial posture was an equilibrium posture, ample time was available to prepare for the jump regardless of whether the trigger was given or not. Perhaps the difference in jump height had to do with the natural tendency to make a small countermovement before starting the push-off in a jump from a squatted position. While the participants made such a small countermovement by 2.0 cm in SFH and by a mere 0.5 cm in SFL, they suppressed this natural tendency in the corresponding ST-jumps (Table 1, see also Panel B4 of Figure 3). During a countermovement, muscles can build up their active state and force, and hence can produce more work over the first part of the range of shortening than during a perfect squat jump in which shortening begins as soon as active state starts to build up (29). Thus, a lower jump height in ST-jumps than in SF-jumps could be due to the performance-enhancing effect of making a small countermovement being absent in the ST-jumps but present in SF-jumps. Evidently, adding to the instruction to “jump as high as possible” the instruction to do this “as soon as possible following the trigger onset” resulted in a loss of jump height, but the loss was small.

Our second finding was that jump height in CT-jumps, in which the posture from which to jump was unknown until trigger onset, was not less but actually greater than in the corresponding ST-jumps, in which the posture was known long before trigger onset; it even tended to be greater than in the corresponding SF-jumps (Table 1). As argued in the Results-section, this too was presumably due to the performance-enhancing effect of a countermovement, even though this movement was made slowly in the experiments. Although effects of the slow countermovement on performance were undesired, there seems to be no indication that performance was submaximal in jumps made as soon as possible following a trigger from a posture that was unknown beforehand.

Our third finding was that muscle activation patterns were posture-specific. Both in ST-jumps and in CT-jumps we found that the onset of *nsrEMG* of hamstrings relative to the onset of *nsrEMG* of gluteus maximus occurred earlier as *z_CoM_* at the bottom posture was lower, and that the onset of *nsrEMG* of m. gastrocnemius relative to the onset of *nsrEMG* of gluteus maximus occurred later as *z_CoM_* at the bottom posture was lower (Columns C and D in Figure 4, Table 2). In a previous study the systematic shift in activation onset of gastrocnemius with respect to that of gluteus maximus had already been found and proven to benefit jump height of a simulation model (9). It seems, therefore, that even in CT-jumps, in which the posture from which to jump was unknown until trigger onset, muscle activation patterns were adapted to the posture at the start of the jump, to the benefit of jump height.

Our fourth finding was that reaction time was not greater in CT-jumps than in ST-jumps (cf. Figure 4 columns A-B, Table 3). We were amazed to find that in CTM and CTL-jumps, the *nsrEMG* onsets of hamstrings and vasti were already released within 200 ms after trigger onset (column B in Figure 4, Table 3). Note also that already within this short period, systematic delays depending on posture occurred between the *nsrEMG* onsets of hamstrings and gluteus maximus (columns B and D in Figure 4, Table 2).

In sum, we have found in CT-jumps that posture-specific muscle activation patterns, resulting in successful jumping performance, may be released within 200 ms following trigger onset. Admittedly, our participants had several years of practice in sports that involved jumping; it would be interesting to investigate if participants who have less practice with jumping also react so quickly and successfully. One question that may be raised with respect to the generation of muscle activation patterns in CT-jumps is what happens before trigger onset. Is the participant merely concentrating on the task of lowering the body while keeping balance on the balls of the feet and toes, or is he already preparing a muscle activation pattern for the upcoming jump? The idea that responses are prepared and kept ready to be released ensues from StartReact studies, in which startle acoustic stimuli are used to involuntarily release prepared responses at different time points preceding an imperative stimulus (30–33). For jumps from an equilibrium posture (SF and ST), it may be conjectured that participants prepare muscle activation patterns and, if necessary, keep them ready to be released at trigger onset. This preparation could theoretically involve mental simulations with an internal model of the musculoskeletal system to find the optimal muscle activation pattern, i.e., the pattern that maximizes jump height from the given initial posture. However, this is not possible for CT-jumps, because the posture from which to jump is not known before trigger onset! Considering that CT-jumps are not lower (Table 1) and do not show larger reaction times (Table 3) than the corresponding ST-jumps, there seems to be no reason to assume that any pre-planning or pre-programming of muscle activation patterns based on mental simulation and optimization occurs before trigger onset in ST-jumps and, by extension, in free jumping.

A final question that may be raised with respect to the generation of muscle activation patterns in CT-jumps is what happens after trigger onset. We have already seen that the first muscles already become active within 200 ms from trigger onset (Table 3), and this reaction time includes the time it takes the auditory trigger to reach the brain and for the signals from the brain to activate the leg muscle fibers. It is interesting to compare this time to the latencies of the startle reflex, in which there is no cortical involvement; the auditory inputs activate the reticular formation and action potentials descend *via* the reticulo-spinal tract to the spinal cord and from there *via* the lower motor neurons to the muscles (34). Latencies of the startle reflex to leg muscles are already on the order of 100–130 ms (35). Consequently, in CT-jumps there is almost no time for any further neural computation to generate motor commands; every additional synaptic transmission takes time. Also, any processing would not be able to take into account the bottom posture, considering the fact that the bottom posture is itself dependent on the muscle activation patterns. After all, when the first muscles are activated, the body still has a downward velocity (Panels A2-A3 in Figure 3) and the bottom posture is not reached until this velocity has been reduced to zero by the forces of the leg muscles. Yet, the muscle activation patterns are posture-specific, so postural feedback must somehow be incorporated. We can only speculate as to how the human nervous system manages to do this. Given that the participants needed only 200 ms to generate successful muscle activation patterns, we may need to think of mechanisms organized at the level of the brain stem or the spinal cord. It has been shown that changes in limb position can regulate intrinsic properties of spinal motoneurons (36). For squat jumps from a static equilibrium posture, a speculative network of spiking neurons could in principle map initial postures to muscle stimulation onsets for successful jumps, albeit not maximally high jumps (37). Such a network could perhaps also generate muscle activation patterns for successful jumps when it is activated during a slow countermovement. However, this is nothing but a parsimonious theory; we have no idea how we could investigate whether such a network exists in the central nervous system, let alone how its settings could be learned. At this point, we are simply left with amazement about the fact that humans can generate muscle stimulation patterns for successful jumps from different initial postures in such a short time.

## Data Availability

The raw data supporting the conclusions of this article will be made available by the authors, without undue reservation.
